# Relative infectiousness of SARS-CoV-2 vaccine breakthrough infections, reinfections, and primary infections

**DOI:** 10.1038/s41467-022-28199-7

**Published:** 2022-01-27

**Authors:** Laith J. Abu-Raddad, Hiam Chemaitelly, Houssein H. Ayoub, Patrick Tang, Peter Coyle, Mohammad R. Hasan, Hadi M. Yassine, Fatiha M. Benslimane, Hebah A. Al-Khatib, Zaina Al-Kanaani, Einas Al-Kuwari, Andrew Jeremijenko, Anvar Hassan Kaleeckal, Ali Nizar Latif, Riyazuddin Mohammad Shaik, Hanan F. Abdul-Rahim, Gheyath K. Nasrallah, Mohamed Ghaith Al-Kuwari, Adeel A. Butt, Hamad Eid Al-Romaihi, Abdullatif Al-Khal, Mohametabd H. Al-Thani, Roberto Bertollini

**Affiliations:** 1 Infectious Disease Epidemiology Group, Weill Cornell Medicine-Qatar, Cornell University, Doha, Qatar; 2 World Health Organization Collaborating Centre for Disease Epidemiology Analytics on HIV/AIDS, Sexually Transmitted Infections, and Viral Hepatitis, Weill Cornell Medicine–Qatar, Cornell University, Qatar Foundation – Education City, Doha, Qatar; 3 Department of Population Health Sciences, Weill Cornell Medicine, Cornell University, New York, NY USA; 4 Department of Public Health, College of Health Sciences, QU Health, Qatar University, Doha, Qatar; 5 Mathematics Program, Department of Mathematics, Statistics, and Physics, College of Arts and Sciences, Qatar University, Doha, Qatar; 6 Department of Pathology, Sidra Medicine, Doha, Qatar; 7 Hamad Medical Corporation, Doha, Qatar; 8 Biomedical Research Center, Member of QU Health, Qatar University, Doha, Qatar; 9 Wellcome-Wolfson Institute for Experimental Medicine, Queens University, Belfast, UK; 10 Department of Biomedical Science, College of Health Sciences, Member of QU Health, Qatar University, Doha, Qatar; 11 College of Health Sciences, QU Health, Qatar University, Doha, Qatar; 12 Primary Health Care Corporation, Doha, Qatar; 13 Ministry of Public Health, Doha, Qatar

**Keywords:** Viral infection, Epidemiology

## Abstract

SARS-CoV-2 breakthrough infections in vaccinated individuals and in those who had a prior infection have been observed globally, but the transmission potential of these infections is unknown. The RT-qPCR cycle threshold (Ct) value is inversely correlated with viral load and culturable virus. Here, we investigate differences in RT-qPCR Ct values across Qatar’s national cohorts of primary infections, reinfections, BNT162b2 (Pfizer-BioNTech) breakthrough infections, and mRNA-1273 (Moderna) breakthrough infections. Our matched-cohort analyses of the randomly diagnosed infections show higher mean Ct value in all cohorts of breakthrough infections compared to the cohort of primary infections in unvaccinated individuals. The Ct value is 1.3 (95% CI: 0.9–1.8) cycles higher for BNT162b2 breakthrough infections, 3.2 (95% CI: 1.9–4.5) cycles higher for mRNA-1273 breakthrough infections, and 4.0 (95% CI: 3.5–4.5) cycles higher for reinfections in unvaccinated individuals. Since Ct value correlates inversely with SARS-CoV-2 infectiousness, these differences imply that vaccine breakthrough infections and reinfections are less infectious than primary infections in unvaccinated individuals. Public health benefits of vaccination may have been underestimated, as COVID-19 vaccines not only protect against acquisition of infection, but also appear to protect against transmission of infection.

## Abstract (web summary)

SARS-CoV-2 breakthrough infections in vaccinated individuals and reinfections in those with prior infection are a public health concern. Here, the authors show that viral loads of vaccine breakthrough infections and reinfections are generally lower than primary infections of unvaccinated individuals, potentially indicating lower infectiousness.

## Introduction

Coronavirus Disease 2019 (COVID-19) vaccines have demonstrated protection against the severe acute respiratory syndrome coronavirus 2 (SARS-CoV-2)^1–3^. However, the efficacy against acquisition of infection is imperfect in that vaccines have an efficacy VE_S_ < 100%, particularly against variants of concern^4–6^. Breakthrough infections in vaccinated individuals have been documented^4–9^, but the transmission potential of these infections is poorly understood. It is conceivable that vaccinated persons who acquire the infection may be less infectious than unvaccinated persons who acquire the infection, as the vaccine-primed immune response may attenuate the natural history of infection, by reducing viral replication and accelerating viral clearance, leading to lower viral load and shorter duration of infection^10,11^. There is evidence that seems to support this hypothesis for SARS-CoV-2 infection^12–14^. Therefore, COVID-19 vaccines may be not only efficacious against acquisition of infection (VE_S_), but also against transmission of infection^10,15,16^, thereby adding an additional efficacy for each vaccine, denoted as VE_I_ and defined as the proportional reduction in infectiousness among those infected but vaccinated, compared to those infected but unvaccinated^10^.

Leveraging the national, federated databases that have captured all SARS-CoV-2 vaccinations and polymerase chain reaction (PCR) testing in Qatar since the start of the epidemic (“Methods”), we investigated the effect of vaccination on infectiousness by comparing SARS-CoV-2 real-time (quantitative) reverse transcription-PCR (RT-qPCR) cycle threshold (Ct) values of individuals infected and fully vaccinated with the values of those infected and unvaccinated. The RT-qPCR Ct value is a measure of the inverse of viral load and correlates strongly with culturable virus;^17^ thus, it can be used as a proxy of SARS-CoV-2 infectiousness^17–21^. We also investigated the effect of prior infection on infectiousness at reinfection by comparing the RT-qPCR Ct values among those reinfected with SARS-CoV-2 with the values among those with primary infection. For standardization of Ct values, the comparisons were conducted using only the RT-qPCR-confirmed infections diagnosed using the TaqPath COVID-19 Combo Kits (Thermo Fisher Scientific, USA^22^) which were used for >85% of all RT-qPCR tests in Qatar^4–6,22^.

**Fig. 1:**
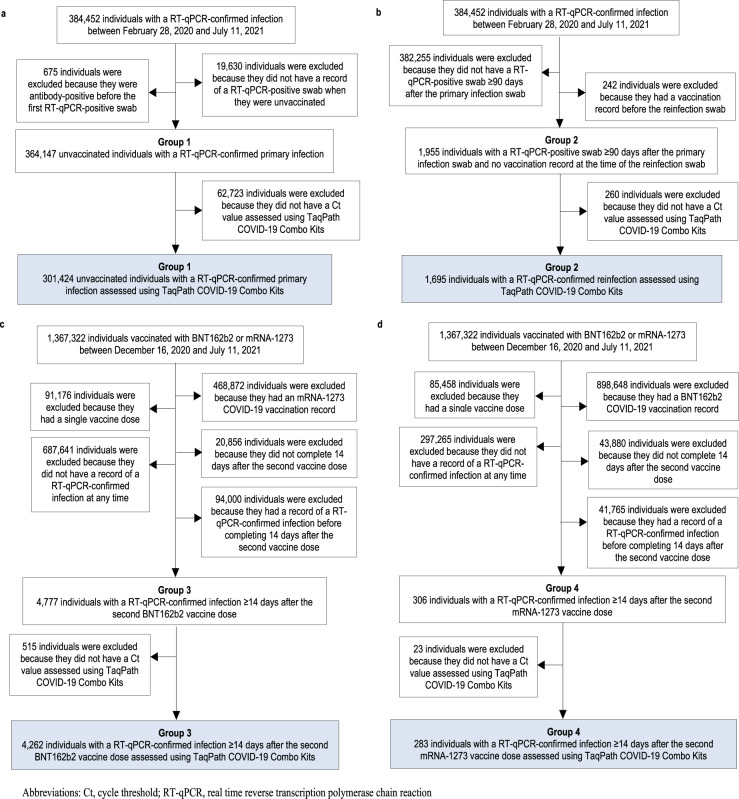
Flowchart illustrating the population selection process. Flowchart illustrating the selection of the cohorts of (**a**) primary infections in unvaccinated individuals, (**b**) reinfections in unvaccinated individuals, (**c**) BNT162b2-vaccine breakthrough infections, and (**d**) mRNA-1273-vaccine breakthrough infections.

The comparisons were implemented utilizing: (i) the national cohort of all 384,452 RT-qPCR-confirmed primary infections since epidemic onset (February 28, 2020) until the end of the study (July 11, 2021; Fig. 1a); (ii) the national cohort of all 1695 RT-qPCR-confirmed reinfections during the same period (Fig. 1b); (iii) the national cohort of all 898,648 individuals vaccinated with the BNT162b2^1^ (Pfizer-BioNTech) vaccine since the first recorded vaccination in Qatar on December 16, 2020 until the end of the study (July 11, 2021; Fig. 1c); and (iv) the national cohort of all 468,872 vaccinated individuals with the mRNA-1273^2^ (Moderna) vaccine during the same period (Fig. 1d).

The BNT162b2 and mRNA-1273 vaccines have been the vaccines of choice in the national immunization campaign in Qatar^4–6,8,9,23,24^. In total, there has been 4777 breakthrough infections in those fully vaccinated with BNT162b2 (0.61% of those fully vaccinated; Fig. 1c) and 306 mRNA-1273 breakthrough infections in those fully vaccinated with mRNA-1273 (0.09% of those fully vaccinated; Fig. 1d). Mass immunization started with the BNT162b2 vaccine, and the mRNA-1273 vaccine was introduced only several weeks later. During this vaccination drive, Qatar experienced two back-to-back epidemic waves dominated by the Alpha^25^ (B.1.1.7) and Beta^25^ (B.1.351) variants, with the Beta wave contributing to most of the diagnosed infections during 2021 (Supplementary Fig. 1)^4–6,8,9,26–28^. While incidence since July 2021 has been dominated by the Delta^25^ (B.1.617.2) variant^4–6,8,9,26–28^, the number of Delta infections remained relatively low with no epidemic wave materializing as of the end date of this study, July 11, 2021.

Primary infection was defined as the first RT-qPCR-positive test for a given individual. Reinfection was defined as the first RT-qPCR-positive test that occurred ≥90 days after the primary infection^29–33^. Breakthrough infection in a vaccinated individual was defined as an RT-qPCR-positive test 14 or more days after the individual received the second vaccine dose, conditional on this RT-qPCR-positive test being the first ever positive for this individual.

## Results

### Study populations

Table 1 shows the Ct value of the RT-qPCR-positive tests stratified by reason for testing for all SARS-CoV-2 infections diagnosed in Qatar using the TaqPath COVID-19 Combo Kits platform from February 28, 2020 to July 11, 2021. Among symptomatic infections, the mean Ct value was 23.1 (95% CI: 23.0–23.1). Among asymptomatic infections, the mean Ct value was 25.2 (95% CI: 25.1–25.2).

**Table 1:** Characteristics of RT-qPCR Ct values in the study population.

Reason for RT-qPCR testing	Setting of RT-qPCR testing	*N*	Proportion (%)	Median	IQR	Mean (95% CI)	SD
Healthcare routine testing^a^	Healthcare facility	21,014	6.8	20.4	16.5–26.0	21.5 (21.4–21.6)	6.2
Clinical suspicion^b^	Healthcare facility	110,948	36.1	22.2	18.0–27.7	23.1 (23.0–23.1)	6.0
Individual request	Healthcare facility	15,489	5.0	24.3	19.1–30.2	24.7 (24.6–24.8)	6.4
Contact tracing	Healthcare facility & community	67,159	21.8	25.0	19.9–30.3	25.1 (25.1–25.2)	6.1
Survey^a^	Community	48,361	15.7	25.8	20.2–31.2	25.6 (25.6–25.7)	6.3
Pre-travel^a^	Healthcare facility	4557	1.5	26.9	20.9–32.0	26.3 (26.2–26.5)	6.4
Port of entry^a^	Healthcare facility	33,287	10.8	28.5	21.0–32.1	26.6 (26.6–26.7)	6.7
Other	Healthcare facility & community	6849	2.2	24.9	19.6–31.0	25.3 (25.1–25.4)	6.4

*Ct* cycle threshold, *IQR* interquartile range, *RT-qPCR* real time reverse transcription polymerase chain reaction, *SD* standard deviation.

^a^An asymptomatic infection was defined as an RT-qPCR-positive test conducted with no prior reason to suspect infection and no reported presence of symptoms compatible with a respiratory tract infection. That is, the RT-qPCR test was conducted as part of a survey (random testing campaigns), for routine healthcare testing, for pre-travel requirement, or at port of entry upon arrival in Qatar.

^b^A symptomatic infection was defined as a RT-qPCR-positive test conducted because of clinical suspicion due to presence of symptoms compatible with a respiratory tract infection.

**Fig. 2:**
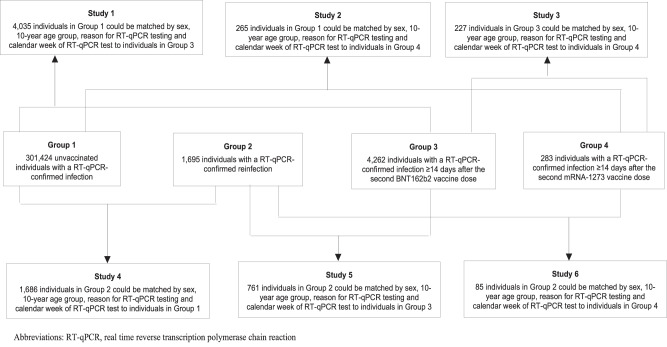
Schematic diagram showing the process of formulating the six pairwise comparisons between study cohorts. Schematic diagram showing the process of formulating the six pairwise comparisons between the cohorts of primary infections in unvaccinated individuals, reinfections in unvaccinated individuals, BNT162b2-vaccine breakthrough infections, and mRNA-1273-vaccine breakthrough infections, after 1:1 matching by sex, 10-year age group, reason for RT-qPCR testing, and calendar week of the RT-qPCR test.

Figure 1 shows the process for identifying eligible primary infections, reinfections, BNT162b2 breakthrough infections, and mRNA-1273 breakthrough infections. Figure 2 schematizes the six pairwise comparisons conducted between these cohorts of infections, after exact matching to the first match in a 1:1 ratio by sex, 10-year age group, reason for RT-qPCR testing, and calendar week of the RT-qPCR test, to control for differences in biology by sex and age, as well as risk of exposure to SARS-CoV-2 in Qatar^34–38^ and variant exposure^4–6,8,9,26–28^ (“Methods”). Only the first RT-qPCR-positive test for each individual for each infection category was used for analysis.

**Fig. 3:**
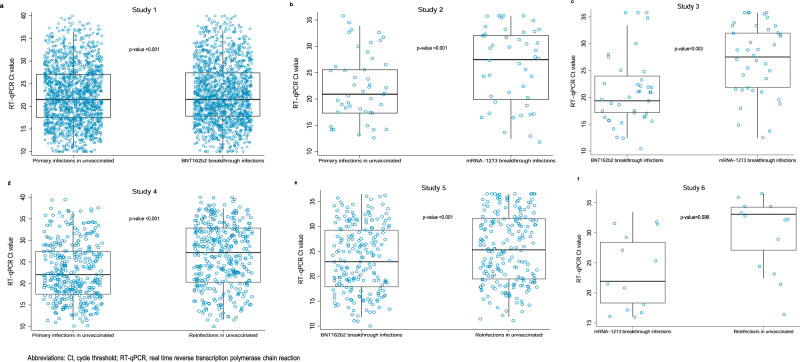
RT-qPCR Ct values in the randomly diagnosed (asymptomatic) SARS-CoV-2 infections. Distribution of these Ct values (blue circles) in the six pairwise comparisons between primary infections in unvaccinated individuals, reinfections in unvaccinated individuals, BNT162b2-vaccine breakthrough infections, and mRNA-1273-vaccine breakthrough infections, **a**–**f**. A randomly diagnosed infection was defined as an RT-qPCR-positive test conducted with no prior reason to suspect infection and no reported presence of symptoms compatible with a respiratory tract infection. That is, the RT-qPCR test was conducted as part of a survey (random testing campaigns), for routine healthcare testing, for pre-travel requirement, or at port of entry upon arrival in Qatar. **a** includes, in each comparison group, *n* = 1584 biologically independent samples, **b** includes *n* = 158 biologically independent samples, **c** includes *n* = 140 biologically independent samples, **d** includes *n* = 987 biologically independent samples, **e** includes *n* = 421 biologically independent samples, and **f** includes *n* = 60 biologically independent samples, each over 1 experiment. Boxplots center lines indicate the median Ct values, box limits indicate the 25% and 75% quartiles, and whiskers indicate maximum and minimum observations within 1.5 of interquartile range. Paired *t* tests were used to compare the difference in means between study groups, with no adjustment for multiple comparisons. Two-sided *p*-values are reported.

Supplementary Tables 1 and 2 show demographic characteristics of the study populations (age, sex, and nationality) in the six pairwise comparisons. Scatter plots of the distribution of RT-qPCR Ct values in each comparison were generated for all RT-qPCR-confirmed infections, regardless of the reason for the RT-qPCR testing (Supplementary Fig. 2), for only the randomly diagnosed (asymptomatic) infections (Fig. 3; “Methods”), and for only the symptomatic infections (Supplementary Fig. 3; “Methods”).

### Differences in RT-qPCR Ct values in all confirmed infections

In the comparisons including all RT-qPCR-confirmed infections, regardless of the reason for the RT-qPCR testing, the mean RT-qPCR Ct value was higher in all cohorts of breakthrough infections compared to the cohort of primary infections in unvaccinated individuals (Table 2 and Supplementary Fig. 2). The Ct value was 1.0 (95% confidence interval (CI): 0.7–1.2) cycles higher for BNT162b2 breakthrough infections, 3.5 (95% CI: 2.5–4.5) cycles higher for mRNA-1273 breakthrough infections, and 3.8 (95% CI: 3.4–4.2) cycles higher for reinfections in unvaccinated individuals. The Ct value was 3.1 (95% CI: 2.0–4.2) cycles higher in mRNA-1273 breakthrough infections than in BNT162b2 breakthrough infections. Compared to reinfections in unvaccinated individuals, the Ct value was 2.1 (95% CI: 0.33–3.8) cycles lower in mRNA-1273 breakthrough infections and 1.7 (95% CI: 1.1–2.3) cycles lower in BNT162b2 breakthrough infections. All differences in Ct values were statistically significant with *p* values ≤ 0.02.

**Table 2:** RT-qPCR Ct values in the six pairwise comparisons including all infections.

Study groups^a^	*N*	Median	Difference in medians	IQR	Mean (95% CI)	SD	Difference in means (95% CI)	*p* value
Study 1								
Primary infections in unvaccinated individuals	4035	23.6		18.5–29.5	24.0 (23.8–24.2)	6.5		
BNT162b2-vaccine breakthrough infections	4035	24.7	1.1	19.2–30.9	25.0 (24.8–25.2)	6.6	1.0 (0.7–1.2)	<0.001
Study 2								
Primary infections in unvaccinated individuals	265	28.9		20.0–33.5	26.8 (25.9–27.6)	7.1		
mRNA-1273-vaccine breakthrough infections	265	32.6	3.7	27.3–34.7	30.3 (29.6–31.0)	5.9	3.5 (2.5–4.5)	<0.001
Study 3								
BNT162b2-vaccine breakthrough infections	227	29.0		20.0–33.5	27.1 (26.2–28.0)	6.0		
mRNA-1273-vaccine breakthrough infections	227	32.6	3.6	27.3–34.7	30.2 (29.4–31.0)	7.0	3.1 (2.0–4.2)	<0.001
Study 4								
Primary infections in unvaccinated individuals	1686	23.6		18.5–29.9	24.2 (23.8–24.5)	6.6		
Reinfections in unvaccinated individuals	1686	29.9	6.3	22.3–33.5	27.9 (27.6–28.3)	6.5	3.8 (3.4–4.2)	<0.001
Study 5								
BNT162b2-vaccine breakthrough infections	761	26.4		20.2–31.9	26.0 (25.5–26.4)	6.5		
Reinfections in unvaccinated individuals	761	29.5	3.1	22.0–33.4	27.7 (27.2–28.1)	6.5	1.7 (1.1–2.3)	<0.001
Study 6								
mRNA-1273-vaccine breakthrough infections	85	31.6		24.1–34.2	28.8 (27.3–30.3)	6.8		
Reinfections in unvaccinated individuals	85	33.0	1.4	29.4–34.4	30.8 (29.7–32.0)	5.3	2.1 (0.3–3.8)	0.022

RT-qPCR Ct values, for all confirmed infections, regardless of the reason for the RT-qPCR testing, in the six pairwise comparisons between primary infections in unvaccinated individuals, reinfections in unvaccinated individuals, BNT162b2-vaccine breakthrough infections, and mRNA-1273-vaccine breakthrough infections. Paired *t*-tests were used to compare the difference in means between study groups, with no adjustment for multiple comparisons. Two-sided *p* values are reported.

*Ct* cycle threshold, *IQR* interquartile range, *RT-qPCR* real-time reverse transcription polymerase chain reaction, *SD* standard deviation.

^a^Study groups were matched in a 1:1 ratio by sex, 10-year age group, reason for RT-qPCR testing, and RT-qPCR test calendar week.

To account for the possibility of prolonged primary infections^39–41^, a comparison was restricted to reinfections in which the RT-qPCR-positive test occurred between 90 and 180 days after the primary infection. Compared to primary infections in unvaccinated individuals, the Ct value was 6.2 (95% CI: 5.4–6.9) cycles higher for the reinfections in unvaccinated individuals (*p* values ≤ 0.001).

The Ct values across the different infection categories were analyzed using matched pairwise comparisons, rather than using an overall regression model, for assessment of pairwise differences, improved statistical precision of the differences, and better control of confounding factors, considering the relatively small number of breakthrough infections in some of the categories. For additional validation of the results, all RT-qPCR-confirmed infections were also analyzed using an overall regression model (Table 3). The results of this analysis were similar and consistent with the results of the main analysis using matched pairwise comparisons.

**Table 3:** Linear regression analyses for the RT-qPCR Ct value across the different categories of infections, adjusting for sex, age, nationality, reason for RT-qPCR testing, and calendar week of RT-qPCR test^a^.

	Original sample size	RT-qPCR Ct value		Univariable regression analysis		Multivariable regression analysis	
	*N* (%)	Mean (SD)	*p* value	β (95% CI)	*p* value	Adjusted *β* (95% CI)	*p* value
Study group							
Primary infections in unvaccinated individuals	301,424 (98.0)	24.4 (6.3)	<0.001	Reference		Reference	
Reinfections in unvaccinated individuals	1695 (0.6)	28.0 (6.4)		3.61 (3.30–3.91)	<0.001	3.67 (3.38–3.96)	<0.001
BNT162b2-vaccine breakthrough infections	4262 (1.4)	24.9 (6.5)		0.60 (0.40–0.79)	<0.001	0.82 (0.63–1.01)	<0.001
mRNA-1273-vaccine breakthrough infections	283 (0.1)	30.2 (6.0)		5.82 (5.08–6.56)	<0.001	3.29 (2.58–4.00)	<0.001
Sex							
Male	220,318 (71.6)	24.6 (6.4)	<0.001	Reference		Reference	
Female	87,346 (28.4)	23.9 (6.2)		−0.61 (−0.66 to −0.56)	<0.001	−0.45 (−0.50 to −0.40)	<0.001
Age group							
20–29 years^b^	67,040 (21.8)	24.7 (6.5)	<0.001	Reference		Reference	
<10 years	21,981 (7.1)	26.4 (5.7)		1.72 (1.63–1.82)	<0.001	2.13 (2.04–2.23)	<0.001
10–19 years	23,581 (7.7)	23.9 (6.0)		−0.80 (-0.90 to −0.71)	<0.001	−0.29 (−0.38 to −0.20)	<0.001
30–39 years	106,493 (34.6)	24.3 (6.5)		−0.39 (−0.45 to −0.33)	<0.001	−0.20 (−0.26 to −0.15)	<0.001
40–49 years	58,072 (18.9)	24.1 (6.3)		−0.60 (−0.67 to −0.53)	<0.001	−0.28 (−0.34 to −0.21)	<0.001
50–59 years	22,299 (7.2)	23.9 (6.2)		−0.78 (−0.87 to −0.68)	<0.001	−0.41 (−0.50 to −0.32)	<0.001
60–69 years	6360 (2.1)	23.5 (6.1)		−1.15 (−1.31 to −0.98)	<0.001	−0.82 (−0.97 to −0.66)	<0.001
70+ years	1838 (0.6)	23.3 (6.0)		−1.38 (−1.67 to −1.09)	<0.001	−1.04 (−1.32 to −0.76)	<0.001
Reason for RT-qPCR testing							
Clinical suspicion	110,948 (36.1)	23.1 (6.0)	<0.001	Reference		Reference	
Pre-travel	4557 (1.5)	26.3 (6.4)		3.26 (3.08–3.44)	<0.001	3.64 (3.46–3.82)	<0.001
Port of entry	33,287 (10.8)	26.6 (6.7)		3.56 (3.49–3.64)	<0.001	3.55 (3.46–3.63)	<0.001
Survey	48,361 (15.7)	25.6 (6.3)		2.56 (2.50–2.63)	<0.001	2.76 (2.69–2.82)	<0.001
Individual request	15,489 (5.0)	24.7 (6.4)		1.66 (1.55–1.76)	<0.001	2.22 (2.11–2.32)	<0.001
Contact tracing	67,159 (21.8)	25.1 (6.1)		2.04 (1.98–2.10)	<0.001	1.70 (1.64–1.76)	<0.001
Healthcare routine testing	21,014 (6.8)	21.5 (6.2)		−1.60 (−1.69 to −1.51)	<0.001	−0.84 (−0.94 to −0.75)	<0.001
Other	6849 (2.2)	25.3 (6.4)		2.21 (2.06–2.36)	<0.001	1.87 (1.71–2.02)	<0.001

*CI* confidence interval, *Ct* cycle threshold, *RT-qPCR* real-time reverse transcription polymerase chain reaction, *SD* standard deviation.

^a^Analyses were conducted on the full sample including all infections diagnosed using TaqPath COVID-19 Combo Kits platform. Categories for calendar week of RT-qPCR test are not presented in the table for brevity.

^b^20–29 years was chosen as a reference category because it is the first fully adult age group.

### Differences in RT-qPCR Ct values in randomly diagnosed infections

In the comparisons including only the randomly diagnosed (asymptomatic) infections (“Methods”), which are perhaps most representative for differences between these cohorts of infections, given the random diagnosis, the mean RT-qPCR Ct value was also higher in all cohorts of breakthrough infections compared to the cohort of primary infections in unvaccinated individuals (Table 4 and Fig. 3). The Ct value was 1.3 (95% CI: 0.9–1.8) cycles higher for BNT162b2 breakthrough infections, 3.2 (95% CI: 1.9–4.5) cycles higher for mRNA-1273 breakthrough infections, and 4.0 (95% CI: 3.5–4.5) cycles higher for reinfections in unvaccinated individuals. The Ct value was 2.2 (95% CI: 0.9–3.6) cycles higher in mRNA-1273 breakthrough infections than in BNT162b2 breakthrough infections. Compared to reinfections in unvaccinated individuals, the Ct value was 2.0 (95% CI: 1.2–2.8) cycles lower in BNT162b2 breakthrough infections and 1.7 (95% CI: −0.3 to 3.7) cycles lower in mRNA-1273 breakthrough infections. All differences in Ct values were statistically significant with *p* values ≤ 0.003 except for the comparison between reinfections and mRNA-1273 breakthrough infections; likely a consequence of the small number of breakthrough infections that have been documented among those vaccinated with the mRNA-1273 vaccine in Qatar.

**Table 4:** RT-qPCR Ct values including only the randomly diagnosed (asymptomatic) infections in the six pairwise comparisons.

Study groups^a^	*N*	Median	Median difference	IQR	Mean (95% CI)	SD	Difference in means (95% CI)	*p* value
Study 1								
Primary infections in unvaccinated individuals	1584	25.8		19.5–31.4	25.5 (25.2–25.8)	6.6		
BNT162b2-vaccine breakthrough infections	1584	27.8	2.0	21.1–32.7	26.8 (26.5–27.2)	6.5	1.3 (0.9–1.8)	<0.001
Study 2								
Primary infections in unvaccinated individuals	158	30.5		23.5–33.7	28.0 (27.0–29.1)	6.7		
mRNA-1273-vaccine breakthrough infections	158	33.3	2.8	29.6–34.8	31.2 (30.4–32.1)	5.5	3.2 (1.9–4.5)	<0.001
Study 3								
BNT162b2-vaccine breakthrough infections	140	31.5		23.9–34.0	28.7 (27.6–29.9)	6.8		
mRNA-1273-vaccine breakthrough infections	140	33.3	1.8	29.3–34.8	31.0 (30.0–32.0)	5.8	2.2 (0.9–3.6)	0.003
Study 4								
Primary infections in unvaccinated individuals	987	25.0		18.6–31.5	24.8 (24.3–25.2)	7.0		
Reinfections in unvaccinated individuals	987	30.9	5.9	24.3–33.8	28.8 (28.4–29.2)	6.2	4.0 (3.5–4.5)	<0.001
Study 5								
BNT162b2-vaccine breakthrough infections	421	28.2		21.1–33.1	27.0 (26.3–27.6)	6.6		
Reinfections in unvaccinated individuals	421	31.2	3.0	24.3–33.9	28.9 (28.3–29.5)	6.2	2.0 (1.2–2.8)	<0.001
Study 6								
mRNA-1273-vaccine breakthrough infections	60	33.1		26.5–34.8	30.0 (28.3–31.7)	6.6		
Reinfections in unvaccinated individuals	60	33.1	0.0	31.1–34.6	31.7 (30.5–32.9)	4.7	1.7 (−0.3 to 3.7)	0.096

RT-qPCR Ct values, including only the randomly diagnosed (asymptomatic) infections, in the six pairwise comparisons between primary infections in unvaccinated individuals, reinfections in unvaccinated individuals, BNT162b2-vaccine breakthrough infections, and mRNA-1273-vaccine breakthrough infections. A randomly diagnosed infection is defined as an RT-qPCR-positive test conducted with no prior reason to suspect infection and no reported presence of symptoms compatible with a respiratory tract infection. Paired *t*-tests were used to compare the difference in means between study groups, with no adjustment for multiple comparisons. Two-sided *p* values are reported.

*Ct* cycle threshold, *IQR* interquartile range, *RT-qPCR* real-time reverse transcription polymerase chain reaction, *SD* standard deviation.

^a^Study groups were matched in a 1:1 ratio by sex, 10-year age group, reason for RT-qPCR testing, and RT-qPCR test calendar week.

### Differences in RT-qPCR Ct values in symptomatic infections

In the comparisons including only the symptomatic infections (“Methods”), the mean RT-qPCR Ct value was also higher in all cohorts of breakthrough infections compared to the cohort of primary infections in unvaccinated individuals, but the difference was smaller for BNT162b2 breakthrough infections (Table 5 and Supplementary Fig. 3). The Ct value was 0.2 (95% CI: −0.2 to 0.6) cycles higher for BNT162b2 breakthrough infections, 4.9 (95% CI: 2.3–7.4) cycles higher for mRNA-1273 breakthrough infections, and 3.8 (95% CI: 2.9–4.7) cycles higher for reinfections in unvaccinated individuals. The Ct value was 5.3 (95% CI: 2.6–8.1) cycles higher in mRNA-1273 breakthrough infections than in BNT162b2 breakthrough infections. Compared to reinfections in unvaccinated individuals, the Ct value was 1.8 (95% CI: 0.6–3.0) cycles lower in BNT162b2 breakthrough infections and 6.4 (95% CI: −0.04 to 12.9) cycles lower in mRNA-1273 breakthrough infections. The differences in Ct values were generally statistically significant with *p* values ≤ 0.05 except for the comparison between primary infections and BNT162b2 breakthrough infections. Notably, very few symptomatic mRNA-1273 breakthrough infections have been documented in Qatar. This may have resulted in the borderline significance in the comparison between mRNA-1273 breakthrough infections and reinfections in unvaccinated individuals (Table 5).

**Table 5:** RT-qPCR Ct values including only the symptomatic infections in the six pairwise comparisons.

Study groups^a^	*N*	Median	Median difference	IQR	Mean (95% CI)	SD	Difference in means (95% CI)	*p* value
Study 1								
Primary infections in unvaccinated individuals	1566	21.5		17.5–31.0	22.5 (22.2–22.8)	6.0		
BNT162b2-vaccine breakthrough infections	1566	21.5	0.0	17.8–27.5	22.7 (22.4–23.0)	6.0	0.2 (−0.2 to 0.6)	0.332
Study 2								
Primary infections in unvaccinated individuals	46	20.9		17.3–25.6	21.7 (20.0–23.3)	5.5		
mRNA-1273-vaccine breakthrough infections	46	27.5	6.6	19.9–32.1	26.6 (24.6–28.6)	6.7	4.9 (2.3–7.4)	<0.001
Study 3								
BNT162b2-vaccine breakthrough infections	39	19.4		17.1–24.0	21.3 (19.3–23.2)	6.0		
mRNA-1273-vaccine breakthrough infections	39	27.5	8.1	21.8–32.0	26.6 (24.5–28.7)	6.5	5.3 (2.6–8.1)	<0.001
Study 4								
Primary infections in unvaccinated individuals	364	22.1		17.5–27.4	22.7 (22.1–23.3)	5.9		
Reinfections in unvaccinated individuals	364	27.2	5.1	20.3–32.9	26.5 (25.8–27.2)	6.8	3.8 (2.9–4.7)	<0.001
Study 5								
BNT162b2-vaccine breakthrough infections	204	22.9		17.8–29.3	23.7 (22.9–24.6)	6.1		
Reinfections in unvaccinated individuals	204	25.3	2.4	19.4–31.6	25.6 (24.6–26.5)	6.6	1.8 (0.6–3.0)	0.003
Study 6								
mRNA-1273-vaccine breakthrough infections	13	21.9		18.3–28.5	23.5 (19.6–27.4)	6.4		
Reinfections in unvaccinated individuals	13	33.1	11.2	27.1–34.3	30.0 (26.2–33.7)	6.3	6.4 (−0.04 to 12.9)	0.051

RT-qPCR Ct values, including only the symptomatic infections, in the six pairwise comparisons between primary infections in unvaccinated individuals, reinfections in unvaccinated individuals, BNT162b2-vaccine breakthrough infections, and mRNA-1273-vaccine breakthrough infections. Paired *t*-tests were used to compare the difference in means between study groups, with no adjustment for multiple comparisons. Two-sided *p* values are reported.

*Ct* cycle threshold, *IQR* interquartile range, *RT-qPCR* real-time reverse transcription polymerase chain reaction, *SD* standard deviation.

^a^Study groups were matched in a 1:1 ratio by sex, 10-year age group, reason for RT-qPCR testing, and RT-qPCR test calendar week.

## Discussion

Breakthrough infections in those vaccinated or who had a prior infection have higher RT-qPCR Ct values than primary infections in unvaccinated persons. While these breakthrough infections are not uncommon, the results indicate that they have lower viral load and are less likely to be infectious than primary infections. While some of these breakthrough infections could lead to secondary transmissions, and indeed some of them did have high viral loads (Fig. 3 and Supplementary Figs. 2 and 3), the risk of onward transmission is reduced, compared to primary infections. Thus, they are of less public health concern.

A consequence of these findings is that the public health benefits of vaccination may be underestimated. In addition to the conventional vaccine efficacy against acquisition of infection (VE_S_), that is assessed in randomized clinical trials^1–3^, there is an additional “breakthrough” efficacy against transmission (VE_I_) that augments the benefits of VE_S_, at least for the BNT162b2 and mRNA-1273 vaccines investigated in this study. The existence of this additional VE_I_ efficacy implies that the reproduction number (*R*_0_) after vaccination is lower than current estimates^10^, and that SARS-CoV-2 incidence may decline faster with vaccine scale-up than previously thought^10^.

One finding of this study is that there appears to be a hierarchy in infectiousness of SARS-CoV-2 infections, where primary infections in unvaccinated persons are most infectious, followed by BNT162b2 breakthrough infections, mRNA-1273 breakthrough infections, and finally reinfections in unvaccinated persons. Strikingly, this hierarchy is the mirror image of the hierarchy observed in the efficacy against acquisition of infection. In our earlier studies on these national cohorts in Qatar, we found that those vaccinated with BNT162b2 had (relatively) the lowest protection against acquisition of infection (at 75% against the Beta variant^4,6,23,42^ that dominated incidence since the onset of vaccination^4–6,8,9,23,24,26–28^). Meanwhile, the protection against acquisition of infection was considerably higher among those vaccinated with mRNA-1273^5,6,42^ and those with a prior infection^29–31,43^. This may indicate that both VE_S_ and VE_I_ are essentially inter-related manifestations of the strength of the vaccine-induced (or natural-infection-induced) immune response. When the immune response is strong against acquisition of infection, it also appears strong at reducing viral replication upon acquisition of the virus, leading to lower viral load and faster infection clearance. Thus, less secondary transmission occurs in breakthrough infections.

While it is well-established that the Ct value correlates inversely with SARS-CoV-2 infectiousness^17–21^, the mathematical form of the relationship between Ct value and transmission risk, or the mathematical form of the relationship between viral load and transmission risk, are inadequately understood^17–21,44–47^. If the transmission risk has a linear decreasing relationship with Ct value, as suggested by a nationwide study from Denmark^18^, the observed differences in Ct value by vaccination and prior infection status may not necessarily translate into large differences in transmission risk. However, if the transmission risk has a linear or a non-linear power-law relationship with viral load, as is the case for the rigorously established transmission risk of HIV infection^48–50^, the observed differences in Ct value would translate into large differences in transmission risk.

This study has limitations. The number of documented mRNA-1273 breakthrough infections was small, thereby limiting the statistical precision of the comparisons involving these infections and leading to estimates with wider 95% CIs; perhaps making them also more prone to bias. The matching further reduced the numbers of infections used in analysis and resulted in different subsets of each category of breakthrough infections being used in the different comparisons. With the relatively small number of breakthrough infections, there was not adequate statistical power to investigate effect modifications by age or other demographic factors. With evidence for waning of vaccine immunity over time after the second dose^8,51–55^, some of the differences observed for those vaccinated with BNT162b2 versus mRNA-1273 may be explained by the shorter duration between the second dose and the breakthrough infection for those mRNA-1273 vaccinated, rather than differences in the biological immunity induced by each of these two vaccines. Future studies could investigate whether RT-qPCR Ct values are lower with increasing time since vaccination or secondary infection.

For symptomatic cases, the date of symptom onset was not available and thus an analysis of the duration between date of symptom onset and date of RT-qPCR testing, for the different categories of infections, was not possible. Only a small proportion of these documented infections was sequenced or RT-qPCR genotyped in Qatar;^4–6,8,9,26–28^ thus, we were unable to implement the above analyses for each variant separately. With the dominance of the Beta variant among cases included in this study (Supplementary Fig. 1), the results are most representative for this variant and may not be representative for the Alpha, Delta or other variants.

The study was implemented on documented infections, but other infections may have occurred and gone undocumented. It is possible that breakthrough infections in those vaccinated or who had a prior infection are less likely to be documented, perhaps because of minimal/mild or no symptoms. However, with the high rate of PCR testing in Qatar, the majority of infections are identified not because of testing symptomatic cases, but because of testing for other reasons, such as random testing campaigns, contact tracing, individual request, routine healthcare testing, pre-travel, and at ports of entry^8^. The results of our study were also consistent when we included only the randomly diagnosed infections.

Imperfect assay sensitivity and specificity of RT-qPCR testing may have affected infection ascertainment, but RT-qPCR testing was performed using a validated commercial platform that has been used globally and has essentially 100% sensitivity and specificity^22^ (“Methods”). Unlike blinded, randomized clinical trials, the investigated observational cohorts were neither blinded nor randomized. Our cohorts predominantly included working-age adults; therefore, results may not necessarily be generalizable to other population groups, such as children or the elderly. Matching was done for sex, age, reason for the RT-qPCR testing, and calendar week of the RT-qPCR test, but could not be done for other factors, such as comorbidities, as these were not available to study investigators. But inclusion of additional factors in the matching would have considerably reduced the sample sizes—breakthrough infections in those vaccinated or who had a prior infection are relatively uncommon. It is noteworthy that matching by age and sex may have served as a proxy for matching by co-morbidity, as comorbidities are associated with older age and may differ between females and males.

In conclusion, prior immunity, whether due to vaccination or prior infection, is associated with lower SARS-CoV-2 viral load upon infection. While breakthrough infections have been observed globally, they appear less infectious than primary infections; thus, they constitute a lesser public health concern. The public health benefits of vaccination are underestimated, as both the BNT162b2 and mRNA-1273 vaccines seem to protect not only against acquisition of infection, but also against transmission of infection. These findings justify optimism and stress the urgency to scale-up vaccination globally in order to robustly control infection transmission and the extent of the pandemic.

## Methods

### Data sources and study design

Analyses were conducted using the centralized, integrated, and standardized national severe acute respiratory syndrome coronavirus 2 (SARS-CoV-2) databases compiled at Hamad Medical Corporation (HMC), the main public healthcare provider and the nationally designated provider for all COVID-19 healthcare needs. Through a nationwide digital health information platform, these databases are complete, and have captured all SARS-CoV-2-related data as well as related demographic details with no missing information since the start of the epidemic, including all records of PCR testing, antibody testing, COVID-19 hospitalizations, vaccinations, infection severity classification per World Health Organization (WHO) guidelines^56^ (performed by trained medical personnel through individual chart reviews), and COVID-19 deaths, also assessed per WHO guidelines^57^.

Every real-time reverse transcription-PCR (RT-qPCR) test conducted in Qatar, regardless of location (outpatient clinic, drive-thru, or hospital, etc.), is classified on the basis of symptoms and the reason for testing (clinical symptoms, contact tracing, random testing campaigns (surveys), individual requests, routine healthcare testing, pre-travel, and port of entry). Qatar has unique demographics by sex and nationality, since expatriates from over 150 countries comprise 89% of the population^34,58^.

The BNT162b2 and mRNA-1273 vaccines have been the vaccines of choice in the national immunization campaign in Qatar^4–6,8,9,23,24^. Nearly all individuals in the cohorts of this study were vaccinated (free of charge) in Qatar, rather than elsewhere. In rare situations where an individual received vaccination outside Qatar, that individual’s vaccination details were still recorded in the health system at the port of entry upon return to Qatar, given the national requirements and to benefit from privileges associated with vaccination, such as quarantine exemption^24^.

Leveraging the national databases, effects of vaccination and of prior infection on SARS-CoV-2 infectiousness were investigated by comparing the RT-qPCR cycle threshold (Ct) values in matched cohorts of primary infections in unvaccinated individuals, reinfections in unvaccinated individuals, BNT162b2 breakthrough infections, and mRNA-1273 breakthrough infections. Only the first RT-qPCR-positive test for each individual for each infection category was used in the analysis. These types of infections are defined in the main text.

In total, six types of pairwise comparisons were conducted on the cohorts of this study, after exact matching to the first match in a 1:1 ratio by sex, 10-year age group, reason for RT-qPCR testing, and calendar week of the RT-qPCR test, to control for differences in biology by sex and age, as well as exposure risk^34–38^ and variant exposure^4–6,8,9,26–28^. It is noteworthy that the first SARS-CoV-2 epidemic wave in Qatar occurred before introduction of any variant of concern and peaked in late May, 2020^34,35^. The second wave was triggered by introduction and expansion of the Alpha^25^ (B.1.1.7) variant and peaked in early March, 2021^4–6,8,9,26–28^. The third wave was dominated by the Beta^25^ (B.1.351) variant, and peaked in the first week of April, 2021^4–6,8,9,26–28^. The Delta^25^ (B.1.617.2) variant has been introduced only recently in Qatar, and as of July 11, 2021, it remains at low incidence^4–6,8,9,26–28^. There is no evidence that any other variant of concern is or has been responsible for appreciable community transmission in Qatar^4–6,8,9,26–28^.

Comparisons across the cohorts of infection were implemented for all RT-qPCR-confirmed infections, for only the symptomatic infections defined as RT-qPCR-positive tests conducted because of clinical suspicion due to symptoms compatible with a respiratory tract infection, and for only the randomly diagnosed (asymptomatic) infections defined as RT-qPCR-positive tests conducted with no prior reason to suspect infection and no reported symptoms compatible with a respiratory tract infection. The latter was strictly defined as an RT-qPCR-positive test conducted as part of a survey (random testing campaigns), for routine healthcare testing, as a pre-travel requirement, or at a port of entry upon arrival in the country^4–6,34^.

All records of RT-qPCR testing in Qatar were examined in this study, but only samples of matched cohorts were included in the analysis. Individuals with a record of a SARS-CoV-2 antibody-positive test before the first RT-qPCR-positive test were excluded from analysis of those with primary infections. Individuals with a record of vaccination before the reinfection diagnosis were excluded from the analysis of those with reinfection. Only breakthrough infections in fully vaccinated individuals were included in the analysis. Being fully vaccinated was defined as completion of ≥14 days after the second dose. Only individuals who had their first and second doses with the same vaccine were included in the analysis.

Reporting of the study followed the STROBE guidelines (Supplementary Table 3).

### Laboratory methods

Nasopharyngeal and/or oropharyngeal swabs were collected for PCR testing and placed in Universal Transport Medium (UTM). Aliquots of UTM were extracted on a QIAsymphony platform (QIAGEN, USA) and tested with RT-qPCR using TaqPath COVID-19 Combo Kits (100% sensitivity and specificity;^22^ Thermo Fisher Scientific, USA) on an ABI 7500 FAST (Thermo Fisher, USA); extracted using a custom protocol^59^ on a Hamilton Microlab STAR (Hamilton, USA) and tested using AccuPower SARS-CoV-2 Real-Time RT-PCR Kits (100% sensitivity and specificity;^60^ Bioneer, Korea) on an ABI 7500 FAST; or loaded directly into a Roche cobas 6800 system and assayed with a cobas SARS-CoV-2 Test (95% sensitivity, 100% specificity^61^; Roche, Switzerland). The first assay targets the viral S, N, and ORF1ab regions. The second targets the viral RdRp and E-gene regions, and the third targets the ORF1ab and E-gene regions.

For standardization of Ct values, only the RT-qPCR-confirmed infections diagnosed using the TaqPath COVID-19 Combo Kits platform (Thermo Fisher Scientific, USA^22^) were included in the analysis. This platform was used for >85% of all RT-qPCR tests in Qatar and reports individual Ct values for each of the N, ORF1ab, and S genes^4–6,22^. The average of these three Ct values was included in the analysis. The correlation between each pair of these Ct values across all RT-qPCR-positive tests was very strong with a Pearson correlation coefficient ≥0.976. In the case of a gene “target failure”, and specifically an S-gene “target failure” that was the defining characteristic of the Alpha cases^62–64^, the average was determined using only the two Ct values of the N and ORF1ab genes.

Antibodies against SARS-CoV-2 in serological samples were detected using a Roche Elecsys Anti-SARS-CoV-2 assay (99.5% sensitivity^65^, 99.8% specificity;^65,66^ Roche, Switzerland), an electrochemiluminescence immunoassay that uses a recombinant protein representing the nucleocapsid (N) antigen for antibody binding. Results were interpreted according to the manufacturer’s instructions (reactive: optical density (proxy for antibody titer^67^) cutoff index ≥1.0 vs. non-reactive: optical density cutoff index <1.0).

All testing was conducted at the HMC Central Laboratory or Sidra Medicine Laboratory, following standardized protocols.

### Statistical analysis

Socio-demographic characteristics of study samples were described using frequency distributions and measures of central tendency. Differences in proportions across categorical variables between study groups were evaluated using Chi-square tests. The distributions of the RT-qPCR Ct values were illustrated using scatter plots and boxplots, and summarized using measures of central tendency and dispersion. Mean differences in the RT-qPCR Ct values between study groups and associated 95% CIs were calculated using paired *t*-tests. Two-sided *p* value of ≤0.05 indicated a significant association.

To provide an additional validation of the results, associations with a higher RT-qPCR Ct value across study groups and matching factors were explored using analysis of variance tests and univariable linear regressions. Beta coefficients (βs), 95% CIs, and two-sided p-values were reported. All covariates were included in the multivariable analysis to estimate the adjusted βs and associated 95% CIs and *p* values. Covariates with *p* value ≤ 0.05 in the multivariable model were considered associated with the RT-qPCR Ct value.

Statistical analyses were conducted in STATA/SE version 17.0^68^.

### Ethical approval

The study was approved by the Hamad Medical Corporation (MRC-05-011) and Weill Cornell Medicine—Qatar (20-00017) Institutional Review Boards with waiver of informed consent.

### Reporting summary

Further information on research design is available in the Nature Research Reporting Summary linked to this article.

## Supplementary information

Supplementary Information

Reporting Summary

## Data Availability

The dataset of this study is a property of the Qatar Ministry of Public Health that was provided to the researchers through a restricted-access agreement that prevents sharing the dataset with a third party or publicly. The data are available under restricted access for confidentiality. Access can be obtained through a direct application for data access to Her Excellency the Minister of Public Health (https://www.moph.gov.qa/english/OurServices/eservices/Pages/Governmental-Health-Communication-Center.aspx). The raw data are protected and are not available due to data privacy laws. Aggregate data are available within the manuscript and its Supplementary information.

## Code availability

Standard epidemiological analyses were conducted using standard commands in STATA/SE 17.0^68^. The commands/code are accessible using URL: https://github.com/IDEGWCMQ/Ct-Infectiousness/blob/main/Code.do; 10.5281/zenodo.5751292^69^.
