# Supplementary material for: Relative infectiousness of SARS-CoV-2 vaccine breakthrough infections, reinfections, and primary infections
Source: Nat Commun. 2022 Jan 27;13:532. doi: 10.1038/s41467-022-28199-7 (PMC8795418; doi:10.1038/s41467-022-28199-7)
Supplement: Supplementary file 1 — Supplementary Information [file 41467_2022_28199_MOESM1_ESM.pdf]

## Supplementary Information

## Table of Contents

**Supplementary Fig. 1. Numbers of daily diagnosed SARS-CoV-2 infections during the study period.** ........ 2

**Supplementary Table 1. Demographic characteristics of the study populations in the three pairwise comparisons between primary infections in unvaccinated individuals, BNT162b2-vaccine breakthrough infections, and mRNA-1273-vaccine breakthrough infections.** ........ 3

**Supplementary Table 2. Demographic characteristics of the study populations in three pairwise comparisons between primary infections in unvaccinated individuals, reinfections in unvaccinated individuals, BNT162b2-vaccine breakthrough infections, and mRNA-1273-vaccine breakthrough infections.** ........ 4

**Supplementary Fig. 2. RT-qPCR Ct values in all confirmed infections, regardless of the reason for the RT-qPCR testing. Distribution of these Ct values (blue circles) in the six pairwise comparisons between primary infections in unvaccinated individuals, reinfections in unvaccinated individuals, BNT162b2-vaccine breakthrough infections, and mRNA-1273-vaccine breakthrough infections, panels 2a-2f. Figure 2a includes, in each comparison group, n=4,035 biologically independent samples, 2b includes n=265 biologically independent samples, 2c includes n=227 biologically independent samples, 2d includes n=1,686 biologically independent samples, 2e includes n=761 biologically independent samples, and 2f includes n=85 biologically independent samples, each over 1 experiment. Boxplots center lines indicate the median Ct values, box limits indicate the 25% and 75% quartiles, and whiskers indicate maximum and minimum observations within 1.5 of interquartile range. Paired t-tests were used to compare the difference in means between study groups, with no adjustment for multiple comparisons. Two-sided p-values are reported.** ........ 5

**Supplementary Fig. 3. RT-qPCR Ct values in the symptomatic SARS-CoV-2 infections. Distribution of these Ct values (blue circles) in the six pairwise comparisons between primary infections in unvaccinated individuals, reinfections in unvaccinated individuals, BNT162b2-vaccine breakthrough infections, and mRNA-1273-vaccine breakthrough infections, panels 3a-3f. A symptomatic infection was defined as an RT-qPCR-positive test conducted because of clinical suspicion due to presence of symptoms compatible with a respiratory tract infection. Figure 3a includes, in each comparison group, n=1,566 biologically independent samples, 3b includes n=46 biologically independent samples, 3c includes n=39 biologically independent samples, 3d includes n=364 biologically independent samples, 3e includes n=204 biologically independent samples, and 3f includes n=13 biologically independent samples, each over 1 experiment. Boxplots center lines indicate the median Ct values, box limits indicate the 25% and 75% quartiles, and whiskers indicate maximum and minimum observations within 1.5 of interquartile range. Paired t-tests were used to compare the difference in means between study groups, with no adjustment for multiple comparisons. Two-sided p-values are reported.** ........ 6

**Supplementary Table 3. STROBE checklist** ........ 7

**Supplementary Fig. 1. Numbers of daily diagnosed SARS-CoV-2 infections during the study period.**

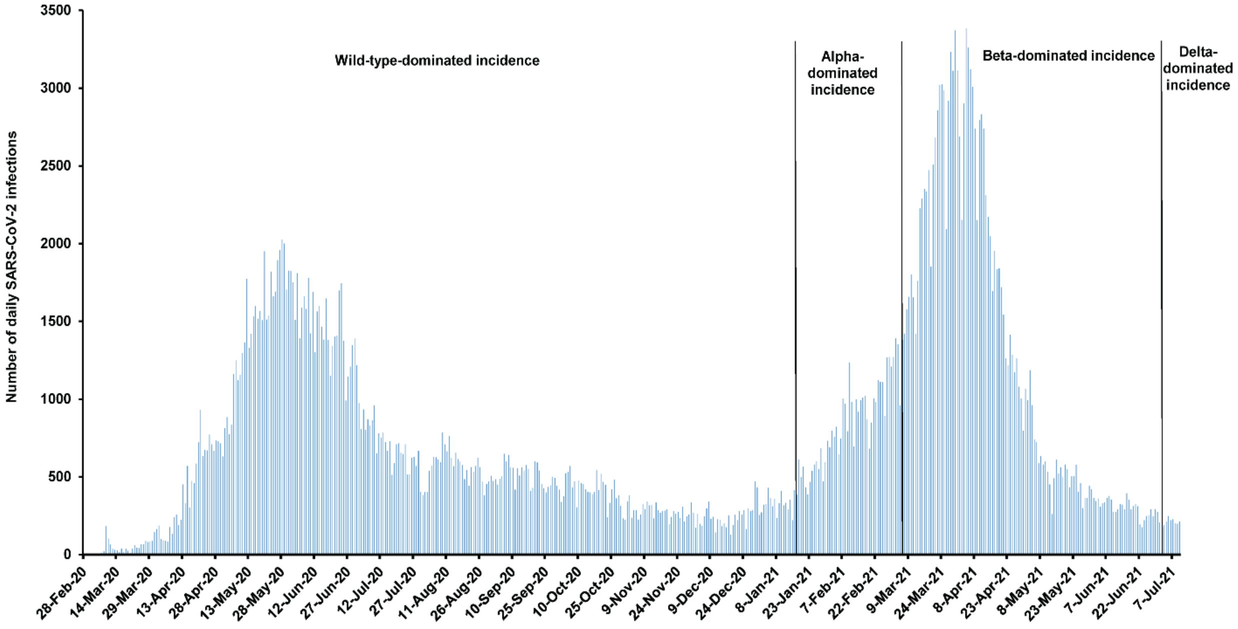

**Supplementary Table 1. Demographic characteristics of the study populations in the three pairwise comparisons between primary infections in unvaccinated individuals, BNT162b2-vaccine breakthrough infections, and mRNA-1273-vaccine breakthrough infections.**

| Characteristics | Study 1* | | | Study 2* | | | Study 3* | | |
|---|---|---|---|---|---|---|---|---|---|
| | Primary infections in unvaccinated individuals | BNT162b2-vaccine breakthrough infections | p-value | Primary infections in unvaccinated individuals | mRNA-1273-vaccine breakthrough infections | p-value | BNT162b2-vaccine breakthrough infections | mRNA-1273-vaccine breakthrough infections | p-value |
| **Median age (IQR)** — years | 41 (34-52) | 42 (34-53) | 0.082 | 35 (30-41) | 35 (30-42) | 0.718 | 36 (31-43) | 36 (31-42) | 0.911 |
| **Age group** — no. (%) | | | | | | | | | |
| <20 years | 53 (1.3) | 53 (1.3) | 1.000 | 3 (1.1) | 3 (1.1) | 1.000 | -- | -- | 1.000 |
| 20-29 years | 439 (10.9) | 439 (10.9) | | 57 (21.5) | 57 (21.5) | | 45 (19.8) | 45 (19.8) | |
| 30-39 years | 1,248 (30.9) | 1,248 (30.9) | | 118 (44.5) | 118 (44.5) | | 97 (42.7) | 97 (42.7) | |
| 40-49 years | 994 (24.6) | 994 (24.6) | | 65 (24.5) | 65 (24.5) | | 62 (27.3) | 62 (27.3) | |
| 50-59 years | 675 (16.7) | 675 (16.7) | | 18 (6.8) | 18 (6.8) | | 20 (8.8) | 20 (8.8) | |
| 60-69 years | 458 (11.4) | 458 (11.4) | | 2 (0.8) | 2 (0.8) | | 2 (0.9) | 2 (0.9) | |
| 70+ years | 168 (4.2) | 168 (4.2) | | 2 (0.8) | 2 (0.8) | | 1 (0.4) | 1 (0.4) | |
| **Sex** | | | | | | | | | |
| Male | 2,526 (62.6) | 2,526 (62.6) | 1.000 | 209 (78.9) | 209 (78.9) | 1.000 | 181 (79.7) | 181 (79.7) | 1.000 |
| Female | 1,509 (37.4) | 1,509 (37.4) | | 56 (21.1) | 56 (21.1) | | 46 (20.3) | 46 (20.3) | |
| **Nationality**† | | | | | | | | | |
| Bangladeshi | 290 (7.2) | 108 (2.7) | <0.001 | 36 (13.6) | 41 (15.5) | <0.001 | 20 (8.8) | 35 (15.4) | <0.001 |
| Egyptian | 149 (3.7) | 321 (8.0) | | 1 (0.4) | 13 (4.9) | | 13 (5.7) | 13 (5.7) | |
| Filipino | 558 (13.8) | 245 (6.1) | | 16 (6.0) | 15 (5.7) | | 7 (3.1) | 12 (5.3) | |
| Indian | 1,173 (29.1) | 855 (21.2) | | 90 (34.0) | 111 (41.9) | | 70 (30.8) | 98 (43.2) | |
| Nepalese | 273 (6.8) | 103 (2.6) | | 24 (9.1) | 8 (3.0) | | 19 (8.4) | 6 (2.6) | |
| Pakistani | 185 (4.6) | 109 (2.7) | | 13 (4.9) | 2 (0.8) | | 2 (0.9) | 2 (0.9) | |
| Qatari | 412 (10.2) | 1,258 (31.2) | | 15 (5.7) | 23 (8.7) | | 54 (23.8) | 17 (7.5) | |
| Sri Lankan | 137 (3.4) | 41 (1.0) | | 5 (1.9) | 16 (6.0) | | 5 (2.2) | 14 (6.2) | |
| Sudanese | 103 (2.6) | 122 (3.0) | | 4 (1.5) | 1 (0.4) | | 5 (2.2) | 1 (0.4) | |
| Other nationalities‡ | 755 (18.7) | 873 (21.6) | | 61 (23.0) | 35 (13.2) | | 32 (14.1) | 29 (12.8) | |

Abbreviations: IQR, interquartile range.
*Study groups were matched in a 1:1 ratio by sex, 10-year age group, reason for RT-qPCR testing, and RT-qPCR test calendar week.
†Nationalities were chosen to represent the most populous groups in the population of Qatar.
‡In Study 1, these comprise: 65 other nationalities in the primary infections and 60 in the BNT162b2-vaccine breakthrough infections; in Study 2: 18 other nationalities in the primary infections and 21 in the mRNA-1273-vaccine breakthrough infections; and in Study 3: 20 other nationalities in the BNT162b2-vaccine breakthrough infections and 16 in the mRNA-1273-vaccine breakthrough infections.

**Supplementary Table 2. Demographic characteristics of the study populations in three pairwise comparisons between primary infections in unvaccinated individuals, reinfections in unvaccinated individuals, BNT162b2-vaccine breakthrough infections, and mRNA-1273-vaccine breakthrough infections.**

| Characteristics | Study 4* | | | Study 5* | | | Study 6* | | |
|---|---|---|---|---|---|---|---|---|---|
| | Primary infections in unvaccinated individuals | Reinfections in unvaccinated individuals | p-value | BNT162b2-vaccine breakthrough infections | Reinfections in unvaccinated individuals | p-value | mRNA-1273-vaccine breakthrough infections | Reinfections in unvaccinated individuals | p-value |
| **Median age (IQR)** — years | 33 (27-40) | 33 (27-40) | 0.703 | 35 (30-42) | 35 (30-41) | 0.290 | 33 (27-38) | 33 (28-37) | 0.818 |
| **Age group** — no. (%) | | | | | | | | | |
| <20 years | 103 (6.1) | 103 (6.1) | 1.000 | 9 (1.2) | 9 (1.2) | 1.000 | 1 (1.2) | 1 (1.2) | 1.000 |
| 20-29 years | 483 (28.7) | 483 (28.7) | | 174 (22.9) | 174 (22.9) | | 27 (31.8) | 27 (31.8) | |
| 30-39 years | 664 (39.4) | 664 (39.4) | | 350 (46.0) | 350 (46.0) | | 41 (48.2) | 41 (48.2) | |
| 40-49 years | 308 (18.3) | 308 (18.3) | | 160 (21.0) | 160 (21.0) | | 12 (14.1) | 12 (14.1) | |
| 50-59 years | 104 (6.2) | 104 (6.2) | | 56 (7.4) | 56 (7.4) | | 3 (3.5) | 3 (3.5) | |
| 60-69 years | 20 (1.2) | 20 (1.2) | | 8 (1.1) | 8 (1.1) | | 1 (1.2) | 1 (1.2) | |
| 70+ years | 4 (0.2) | 4 (0.2) | | 4 (0.5) | 4 (0.5) | | -- | -- | |
| **Sex** | | | | | | | | | |
| Male | 1,434 (85.1) | 1,434 (85.1) | 1.000 | 648 (85.2) | 648 (85.2) | 1.000 | 68 (80.0) | 68 (80.0) | 1.000 |
| Female | 252 (15.0) | 252 (15.0) | | 113 (14.9) | 113 (14.9) | | 17 (20.0) | 17 (20.0) | |
| **Nationality**† | | | | | | | | | |
| Bangladeshi | 168 (10.0) | 178 (10.6) | <0.001 | 133 (17.5) | 73 (9.6) | <0.001 | 12 (14.1) | 12 (14.1) | 0.001 |
| Egyptian | 67 (4.0) | 62 (3.7) | | 41 (5.4) | 25 (3.3) | | 4 (4.7) | 1 (1.2) | |
| Filipino | 138 (8.2) | 90 (5.3) | | 42 (5.4) | 54 (7.1) | | 4 (4.7) | 3 (3.5) | |
| Indian | 572 (33.9) | 521 (30.9) | | 216 (28.4) | 269 (35.4) | | 40 (47.1) | 30 (35.3) | |
| Nepalese | 193 (11.5) | 324 (19.2) | | 31 (4.1) | 137 (18.0) | | 1 (1.2) | 11 (12.9) | |
| Pakistani | 74 (4.4) | 74 (4.4) | | 14 (1.8) | 33 (4.3) | | 1 (1.2) | 10 (11.8) | |
| Qatari | 111 (6.6) | 163 (9.7) | | 229 (30.1) | 44 (5.8) | | 9 (10.6) | 2 (2.4) | |
| Sri Lankan | 75 (4.5) | 48 (2.9) | | 13 (1.7) | 26 (3.4) | | 6 (7.1) | 2 (2.4) | |
| Sudanese | 37 (2.2) | 38 (2.3) | | 19 (2.5) | 16 (2.1) | | 1 (1.2) | 1 (1.2) | |
| Other nationalities‡ | 251 (14.9) | 188 (11.2) | | 133 (17.5) | 73 (9.6) | | 7 (8.2) | 13 (15.3) | |

Abbreviations: IQR, interquartile range.
*Study groups were matched in a 1:1 ratio by sex, 10-year age group, reason for RT-qPCR testing, and RT-qPCR test calendar week.
†Nationalities were chosen to represent the most populous groups in the population of Qatar.
‡In Study 4, these comprise: 48 other nationalities in the primary infections and 37 in the reinfections; in Study 5: 28 other nationalities in the BNT162b2-vaccine breakthrough infections and 31 in the reinfections; and in Study 6: 10 other nationalities in the mRNA-1273-vaccine breakthrough infections and 6 in the reinfections.

**Supplementary Fig. 2. RT-qPCR Ct values in all confirmed infections, regardless of the reason for the RT-qPCR testing. Distribution of these Ct values (blue circles) in the six pairwise comparisons between primary infections in unvaccinated individuals, reinfections in unvaccinated individuals, BNT162b2-vaccine breakthrough infections, and mRNA-1273-vaccine breakthrough infections, panels 2a-2f. Figure 2a includes, in each comparison group, n=4,035 biologically independent samples, 2b includes n=265 biologically independent samples, 2c includes n=227 biologically independent samples, 2d includes n=1,686 biologically independent samples, 2e includes n=761 biologically independent samples, and 2f includes n=85 biologically independent samples, each over 1 experiment. Boxplots center lines indicate the median Ct values, box limits indicate the 25% and 75% quartiles, and whiskers indicate maximum and minimum observations within 1.5 of interquartile range. Paired t-tests were used to compare the difference in means between study groups, with no adjustment for multiple comparisons. Two-sided p-values are reported.**

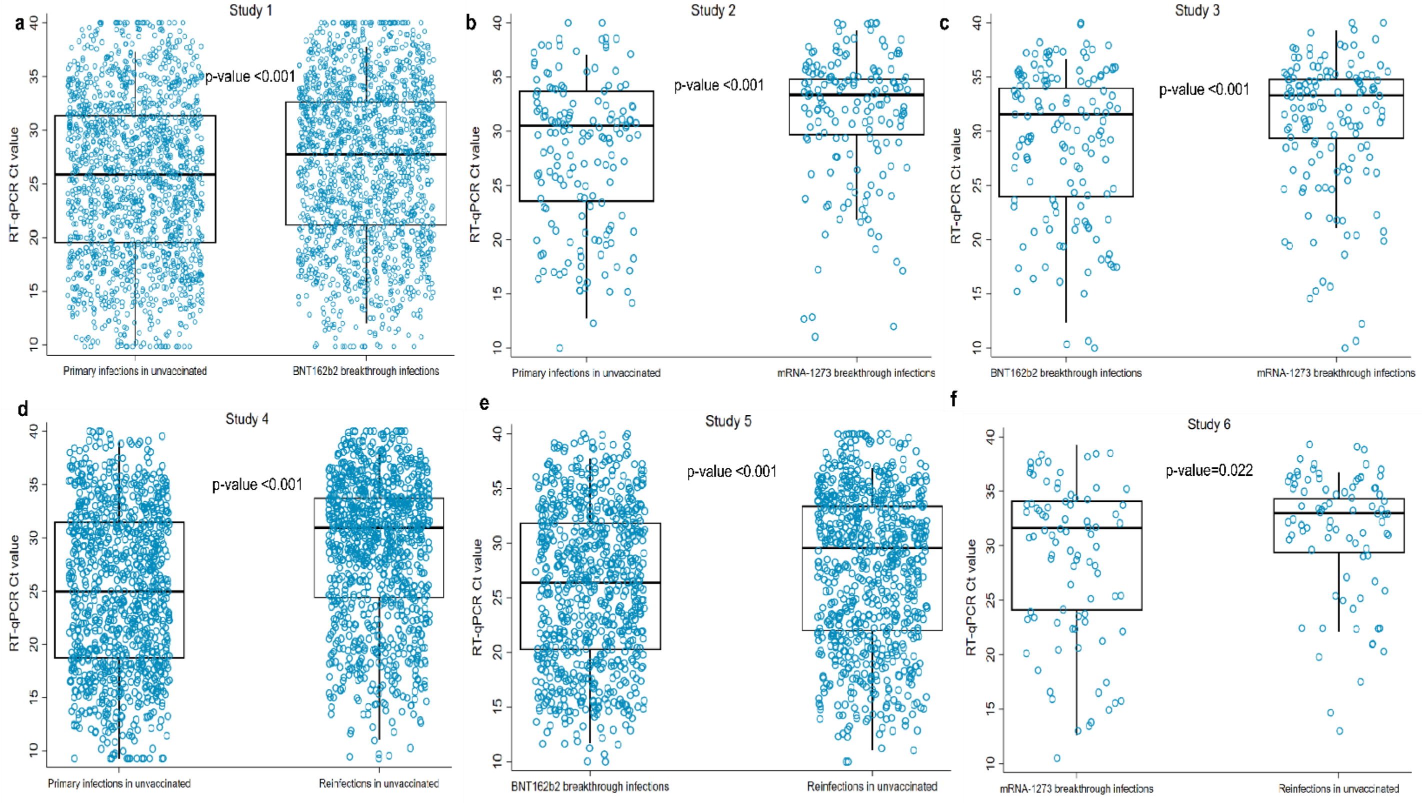


Abbreviations: Ct, cycle threshold; RT-qPCR, real time reverse transcription polymerase chain reaction

**Supplementary Fig. 3. RT-qPCR Ct values in the symptomatic SARS-CoV-2 infections. Distribution of these Ct values (blue circles) in the six pairwise comparisons between primary infections in unvaccinated individuals, reinfections in unvaccinated individuals, BNT162b2-vaccine breakthrough infections, and mRNA-1273-vaccine breakthrough infections, panels 3a-3f. A symptomatic infection was defined as an RT-qPCR-positive test conducted because of clinical suspicion due to presence of symptoms compatible with a respiratory tract infection. Figure 3a includes, in each comparison group, n=1,566 biologically independent samples, 3b includes n=46 biologically independent samples, 3c includes n=39 biologically independent samples, 3d includes n=364 biologically independent samples, 3e includes n=204 biologically independent samples, and 3f includes n=13 biologically independent samples, each over 1 experiment. Boxplots center lines indicate the median Ct values, box limits indicate the 25% and 75% quartiles, and whiskers indicate maximum and minimum observations within 1.5 of interquartile range. Paired t-tests were used to compare the difference in means between study groups, with no adjustment for multiple comparisons. Two-sided p-values are reported.**

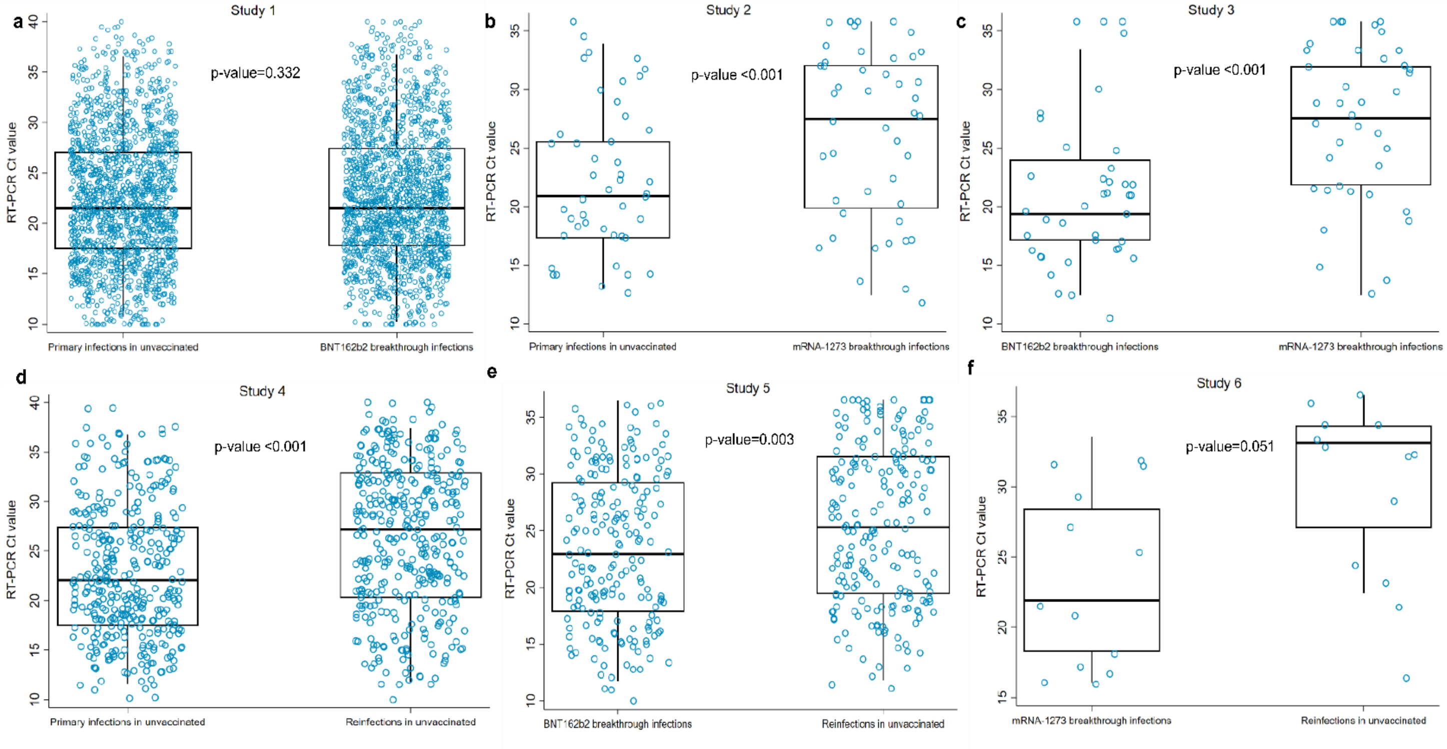


Abbreviations: Ct, cycle threshold; RT-qPCR, real time reverse transcription polymerase chain reaction

## Supplementary Table 3. STROBE checklist.

| | Item No | Recommendation | Main Text |
|---|---|---|---|
| **Title and abstract** | 1 | (*a*) Indicate the study's design with a commonly used term in the title or the abstract | Abstract |
| | | (*b*) Provide in the abstract an informative and balanced summary of what was done and what was found | Abstract |
| **Introduction** | | | |
| Background/rationale | 2 | Explain the scientific background and rationale for the investigation being reported | Introduction paragraphs 1-4 |
| Objectives | 3 | State specific objectives, including any prespecified hypotheses | Introduction paragraphs 3 & 5 |
| **Methods** | | | |
| Study design | 4 | Present key elements of study design early in the paper | Methods ('Data sources and study design') |
| Setting | 5 | Describe the setting, locations, and relevant dates, including periods of recruitment, exposure, follow-up, and data collection | Methods ('Data sources and study design') & Figure 1 |
| Participants | 6 | (*a*) Give the eligibility criteria, and the sources and methods of selection of participants | Methods ('Data sources and study design') & Figure 1 |
| Variables | 7 | Clearly define all outcomes, exposures, predictors, potential confounders, and effect modifiers. Give diagnostic criteria, if applicable | Methods |
| Data sources/ measurement | 8* | For each variable of interest, give sources of data and details of methods of assessment (measurement). Describe comparability of assessment methods if there is more than one group | Methods ('Data sources and study design' & 'Statistical analysis') & Supp. Tables 1 & 2 |
| Bias | 9 | Describe any efforts to address potential sources of bias | Methods ('Data sources and study design' & 'Statistical analysis') & Table 3 |
| Study size | 10 | Explain how the study size was arrived at | Methods ('Data sources and study design') & Figures 1-2 |
| Quantitative variables | 11 | Explain how quantitative variables were handled in the analyses. If applicable, describe which groupings were chosen and why | Methods ('Statistical analysis') & Supp. Tables 1 & 2 |
| Statistical methods | 12 | (*a*) Describe all statistical methods, including those used to control for confounding | Methods ('Statistical analysis') |
| | | (*b*) Describe any methods used to examine subgroups and interactions | Methods ('Statistical analysis') |
| | | (*c*) Explain how missing data were addressed | NA, see Methods ('Data sources and study design') |
| | | (*d*) If applicable, describe analytical methods taking account of sampling strategy | Methods ('Statistical analysis') |
| | | (*e*) Describe any sensitivity analyses | Methods ('Statistical analysis') & Table 3 |
| **Results** | | | |
| Participants | 13* | (a) Report numbers of individuals at each stage of study—eg numbers potentially eligible, examined for eligibility, confirmed eligible, included in the study, completing follow-up, and analysed | Results ('Study populations') & Figures 1-2 |
| | | (b) Give reasons for non-participation at each stage | Figures 1-2 |
| | | (c) Consider use of a flow diagram | Figures 1-2 |
| Descriptive data | 14* | (a) Give characteristics of study participants (eg demographic, clinical, social) and information on exposures and potential confounders | Supp. Tables 1 & 2 |

| | | | |
|---|---|---|---|
| | | (b) Indicate number of participants with missing data for each variable of interest | NA, see Methods ('Data sources and study design') |
| Outcome data | 15* | Report numbers of outcome events or summary measures | Results ('Differences in RT-qPCR Ct values in all confirmed infections' to 'Differences in RT-qPCR Ct values in symptomatic infections', & Tables 2-5 |
| Main results | 16 | (*a*) Give unadjusted estimates and, if applicable, confounder-adjusted estimates and their precision (eg, 95% confidence interval). Make clear which confounders were adjusted for and why they were included | Results ('Differences in RT-qPCR Ct values in all confirmed infections' to 'Differences in RT-qPCR Ct values in symptomatic infections', Figure 3, & Tables 2-5 |
| | | (*b*) Report category boundaries when continuous variables were categorized | Supp. Tables 1 & 2 |
| | | (*c*) If relevant, consider translating estimates of relative risk into absolute risk for a meaningful time period | NA |
| Other analyses | 17 | Report other analyses done—eg analyses of subgroups and interactions, and sensitivity analyses | Supplementary Figs. 2 & 3 & Tables 3-5 |
| **Discussion** | | | |
| Key results | 18 | Summarise key results with reference to study objectives | Discussion paragraphs 1-4 |
| Limitations | 19 | Discuss limitations of the study, taking into account sources of potential bias or imprecision. Discuss both direction and magnitude of any potential bias | Discussion paragraphs 5-8 |
| Interpretation | 20 | Give a cautious overall interpretation of results considering objectives, limitations, multiplicity of analyses, results from similar studies, and other relevant evidence | Discussion paragraph 9 |
| Generalisability | 21 | Discuss the generalisability (external validity) of the study results | Discussion paragraph 8 |
| **Other information** | | | |
| Funding | 22 | Give the source of funding and the role of the funders for the present study and, if applicable, for the original study on which the present article is based | Acknowledgements |

Abbreviations: NA, not applicable; Supp, Supplementary Information.
