# Supplementary material for: Relative infectiousness of SARS-CoV-2 vaccine breakthrough infections, reinfections, and primary infections
Source: Nat Commun. 2022 Jan 27;13:532. doi: 10.1038/s41467-022-28199-7 (PMC8795418; doi:10.1038/s41467-022-28199-7)
Supplement: Supplementary file 2 — Reporting Summary [file 41467_2022_28199_MOESM2_ESM.pdf]

nature research

Corresponding author(s): Laith J Abu-Raddad

Last updated by author(s): December 2, 2021

Reporting Summary

Nature Research wishes to improve the reproducibility of the work that we publish. This form provides structure for consistency and transparency in reporting. For further information on Nature Research policies, see our Editorial Policies and the Editorial Policy Checklist.

Statistics

For all statistical analyses, confirm that the following items are present in the figure legend, table legend, main text, or Methods section.

n/a	Confirmed	
☐	☒	The exact sample size (*n*) for each experimental group/condition, given as a discrete number and unit of measurement
☐	☒	A statement on whether measurements were taken from distinct samples or whether the same sample was measured repeatedly
☐	☒	The statistical test(s) used AND whether they are one- or two-sided<br>*Only common tests should be described solely by name; describe more complex techniques in the Methods section.*
☐	☒	A description of all covariates tested
☐	☒	A description of any assumptions or corrections, such as tests of normality and adjustment for multiple comparisons
☐	☒	A full description of the statistical parameters including central tendency (e.g. means) or other basic estimates (e.g. regression coefficient) AND variation (e.g. standard deviation) or associated estimates of uncertainty (e.g. confidence intervals)
☐	☒	For null hypothesis testing, the test statistic (e.g. *F*, *t*, *r*) with confidence intervals, effect sizes, degrees of freedom and *P* value noted<br>*Give P values as exact values whenever suitable.*
☒	☐	For Bayesian analysis, information on the choice of priors and Markov chain Monte Carlo settings
☒	☐	For hierarchical and complex designs, identification of the appropriate level for tests and full reporting of outcomes
☐	☒	Estimates of effect sizes (e.g. Cohen's *d*, Pearson's *r*), indicating how they were calculated

Our web collection on statistics for biologists contains articles on many of the points above.

Software and code

Policy information about availability of computer code

Data collection	Not applicable
Data analysis	Analyses were conducted in STATA/SE 17.0. The commands/code are accessible using URL: https://github.com/IDEGWCMQ/Ct-Infectiousness/blob/main/Code.do

For manuscripts utilizing custom algorithms or software that are central to the research but not yet described in published literature, software must be made available to editors and reviewers. We strongly encourage code deposition in a community repository (e.g. GitHub). See the Nature Research guidelines for submitting code & software for further information.

Data

Policy information about availability of data

All manuscripts must include a data availability statement. This statement should provide the following information, where applicable:

- Accession codes, unique identifiers, or web links for publicly available datasets
- A list of figures that have associated raw data
- A description of any restrictions on data availability

The dataset of this study is a property of the Qatar Ministry of Public Health that was provided to the researchers through a restricted-access agreement that prevents sharing the dataset with a third party or publicly. The data are available under restricted access for confidentiality. Access can be obtained through a direct application for data access to Her Excellency the Minister of Public Health (https://www.moph.gov.qa/english/OurServices/eservices/Pages/Governmental-Health-Communication-Center.aspx). The raw data are protected and are not available due to data privacy laws. Aggregate data are available within the manuscript and its Supplementary information.

Field-specific reporting

Please select the one below that is the best fit for your research. If you are not sure, read the appropriate sections before making your selection.

☒ Life sciences ☐ Behavioural & social sciences ☐ Ecological, evolutionary & environmental sciences

For a reference copy of the document with all sections, see nature.com/documents/nr-reporting-summary-flat.pdf

Life sciences study design

All studies must disclose on these points even when the disclosure is negative.

Sample size	Coronavirus Disease 2019 (COVID-19) laboratory testing, vaccination, clinical infection data, and related demographic details were extracted from the integrated nationwide digital-health information platform that hosts the national, federated SARS-CoV-2 databases. These databases are complete and have captured all SARS-CoV-2-related data since epidemic onset. The data are based on two national cohorts. The first includes individuals tested using RT-qPCR in Qatar, while the second includes vaccinated individuals in Qatar. Sample size varied depending on the definition used for study group. Primary infection was defined as the first RT-qPCR-positive test for a given individual. Reinfection was defined as the first RT-qPCR-positive test that occurred ≥90 days after the primary infection. Breakthrough infection in a vaccinated individual was defined as an RT-qPCR-positive test 14 or more days after the individual received the second vaccine dose of either BNT162b2 or mRNA-1273, conditional on this RT-qPCR-positive test being the first ever positive for this individual. Groups were matched one-to-one by sex, 10-year age group, reason for SARS-CoV-2 RT-qPCR testing, and calendar week of RT-qPCR test. Only matched cases and controls were included in the analysis. Given that the sample sizes were based on national cohorts with only individuals that do not fit the eligibility criteria excluded, the sample size for each sub-study can be considered sufficient. Detailed sample sizes can be found in Figures 1-2.
Data exclusions	Exclusion criteria were specified in each study group a priori and are detailed in Figures 1-2. Only individuals with an RT-qPCR-confirmed infection tested using the TaqPath COVID-19 Combo Kits platform were eligible for inclusion in the analysis. RT-qPCR-positive tests within 90 days of a primary infection were excluded. Similarly, RT-qPCR-positive tests within 14 days after administration of the second vaccine dose (for either BNT162b2 or mRNA-1273) were excluded. Only the first RT-qPCR-positive test for each individual for each infection category was used in analysis after applying these exclusions.
Replication	For replication, additional analyses were conducted to estimate associations with RT-qPCR cycle threshold (Ct) value after 1) comparing all study groups while adjusting for matching factors in linear regression analyses, 2) restricting the analysis to either symptomatic infection (defined as an RT-qPCR-positive test conducted because of clinical suspicion due to presence of symptoms compatible with a respiratory tract infection) or asymptomatic infection (defined as an RT-qPCR-positive test conducted with no reported presence of symptoms compatible with a respiratory tract infection), and 3) restricting the analysis for reinfections that occurred within 90-180 days after the primary infection. These analyses were performed only once independently. All analyses confirmed/reproduced estimates of pairwise differences in Ct values between groups obtained in the main analysis.
Randomization	Not applicable as this is an observational study where individuals are aware of both their infection status and their vaccination status. However, to ensure control of confounding, study groups were matched one-to-one by sex, 10-year age group, reason for SARS-CoV-2 RT-qPCR testing, and calendar week of RT-qPCR test. To ensure that estimates of pairwise differences in Ct values were not biased, a validation analysis was conducted by assessing differences in Ct values across all study groups in linear regression analyses while adjusting for matching factors .
Blinding	Not applicable as this is an observational study where individuals are aware of both their infection status and their vaccination status.

Reporting for specific materials, systems and methods

We require information from authors about some types of materials, experimental systems and methods used in many studies. Here, indicate whether each material, system or method listed is relevant to your study. If you are not sure if a list item applies to your research, read the appropriate section before selecting a response.

Materials & experimental systems

n/a	Involved in the study	
☒	☐	Antibodies
☒	☐	Eukaryotic cell lines
☒	☐	Palaeontology and archaeology
☒	☐	Animals and other organisms
☐	☒	Human research participants
☒	☐	Clinical data
☒	☐	Dual use research of concern

Methods

n/a	Involved in the study	
☒	☐	ChIP-seq
☒	☐	Flow cytometry
☒	☐	MRI-based neuroimaging

Human research participants

Policy information about studies involving human research participants

Population characteristics	The demographic characteristics of the different study populations can be found in Supplementary Tables 1 and 2.
Recruitment	This is a retrospective study where COVID-19 laboratory testing, vaccination, clinical infection data, and related demographic details were extracted from the integrated nationwide digital-health information platform that hosts the national, federated SARS-CoV-2 databases. These databases are complete with no missing information for RT-qPCR testing, COVID-19 vaccinations, COVID-19 hospitalizations, and basic demographic details, and have captured all SARS-CoV-2-related data since epidemic onset. Study groups were defined based on analysis on these data. Study groups were determined based on presence of 1) primary infection defined as the first RT-qPCR-positive test for a given individual; 2) reinfection defined as the first RT-qPCR-positive test that occurred ≥90 days after the primary infection; 3) breakthrough infection in a vaccinated individual defined as an RT-qPCR-positive test 14 or more days after the individual received the second vaccine dose of either BNT162b2 or mRNA-1273, conditional on this RT-qPCR-positive test being the first ever positive for this individual.
Ethics oversight	The study was approved by the Hamad Medical Corporation and Weill Cornell Medicine-Qatar Institutional Review Boards with waiver of informed consent.

Note that full information on the approval of the study protocol must also be provided in the manuscript.
